# Bioinformatic Characterization and Functional Analysis of a Lipid Transfer Protein from *Panax ginseng* Involved in Biotic and Abiotic Stress Responses

**DOI:** 10.3390/ijms27167094

**Published:** 2026-08-07

**Authors:** Tianxia Sun, Zhimei Liu, Qingbin Liu, Heng Li, Miao Zhang, Ge Hui, Yu Zhao

**Affiliations:** Jilin Ginseng Academy, Changchun University of Chinese Medicine, Changchun 130117, China; 15844014726@163.com (T.S.); 15843048017@163.com (Z.L.); lqbin2000@sina.com (Q.L.); liheng0324@163.com (H.L.); zmiao0606@163.com (M.Z.)

**Keywords:** LTP, *Panax ginseng*, bioinformatics analysis, drought stress, biological stress

## Abstract

Plant lipid transfer proteins (LTPs) are key components in defense against biotic and abiotic stresses, yet their functional diversity in *Panax ginseng* remains unclear. This study aimed to characterize LTP and evaluate its role in stress tolerance. The gene was identified from ginseng transcriptome data and analyzed using bioinformatics tools to determine its structural and physicochemical properties. *Panax ginseng *lipid transfer protein (*PgLTP*) was then heterologously expressed in *Arabidopsis thaliana *(*A. thaliana*). Transgenic lines were evaluated under fungal infection, drought, and salt stress conditions. Physiological and molecular responses, including reactive oxygen species (ROS) accumulation, malondialdehyde (MDA) content, proline levels, electrolyte leakage, and stomatal behavior under abscisic acid (ABA) treatment, were assessed. Bioinformatic analysis indicated that *PgLTP* encodes a small protein of approximately 12 kDa containing ten conserved cysteine residues, four α-helices, a signal peptide, and a transmembrane region, suggesting structural divergence from typical LTPs. Functional assays showed that transgenic plants exhibited significantly reduced disease indices under *Fusarium oxysporum* (*F*.* oxysporum*) and *Cylindrocarpon destructans* (*C*.* destructans*) infection. Under drought and salinity stress, transgenic lines demonstrated higher germination and survival rates, enhanced proline accumulation, reduced oxidative damage, and lower electrolyte leakage compared with the wild type. Additionally, *PgLTP* exhibited enhanced ABA-responsive stomatal closure, which was associated with reduced water loss. These findings indicate that PgLTP contributes to plant tolerance against both biotic and abiotic stresses, which is associated with changes in redox status, osmotic adjustment capacity, and stomatal responses.

## 1. Introduction

The plant is an open system, and the factors that harm plants in the natural environment are referred to as stresses; these stresses include both biotic and abiotic stresses. Biotic stresses include diseases, pests, and weeds, and abiotic stresses include cold, heat, drought, and salinity. Plant stress resistance refers to the ability of plants to adapt to and withstand adverse environments, including both biotic and abiotic stresses. Plants produce defense mechanisms to resist external stimuli and can better adapt to the environment through a series of physiological and biochemical changes, such as adaptive changes in tissue and organ phenotypes, accumulation of osmotic substances, enhancement of antioxidant enzyme activities, and accumulation of disease resistance-related proteins [1,2,3,4,5].

Plant lipid transfer proteins (LTPs) are a class of small proteins, typically ranging from 7 to 10 kDa in size. A hallmark of their structure is the presence of a highly conserved eight-cysteine motif (8CM), which forms four stabilizing disulfide bonds. These proteins generally adopt a structure composed of four to five α-helices, creating a characteristic hydrophobic cavity [6]. Earlier classification efforts distinguished LTPs primarily based on molecular weight and structural features, categorizing them as LTP1 and LTP2 [7]. However, more recent approaches have introduced refined criteria that include intron positioning, spacing between cysteine residues, sequence similarity, and post-translational modifications. On this basis, LTPs have been grouped into five principal types with four secondary forms [8,9].

LTPs are broadly distributed throughout plant tissues and are implicated in numerous physiological processes, including cellular defense, signal communication, and metabolic pathway regulation [10]. In recent years, there has been growing interest in the involvement of LTPs in plant responses to environmental stresses, both biotic including fungal infections and insect attacks, and abiotic, including salinity, drought, and temperature extremes.

LTPs have been found to contribute to biotic stress responses through several distinct mechanisms: compromising pathogen cell membrane integrity, thereby suppressing their proliferation; encouraging cuticular wax deposition to hinder pathogen entry; initiating systemic resistance responses; modulating stomatal closure to prevent ingress by pathogens such as Phytophthora; competitively interfering with elicitor perception; dampening overactive immune reactions; and participating in signaling related to symbiotic plant–microbe associations [11].

LTPs have been associated with abiotic stress responses by maintaining intracellular balances. They may function by regulating reactive oxygen species (ROS) levels to preserve redox homeostasis, interacting with hormone signaling cascades, contributing to the upkeep of cell wall architecture, and stabilizing cellular membranes under adverse conditions [12]. Ginseng has gradually formed complex stress resistance mechanisms to cope with various unfavorable factors in the environment during the long-term evolutionary process. Investigation into and application of the stress resistance mechanisms of ginseng are essential for improving the quality and yield of medicinal herbs.

## 2. Results

### 2.1. Bioinformatics Analysis of PgLTP

Sequencing of the purified product revealed a 363 bp sequence encoding 120 amino acids (see Supplementary Figure S1 online). Sequence analysis revealed that PgLTP contained 10 cysteine residues(see Supplementary Figure S2 online). Two cysteine residues (Cys16 and Cys20) were located within the N-terminal signal peptide, whereas the remaining eight cysteine residues (Cys32, Cys44, Cys53, Cys54, Cys72, Cys82, Cys91, and Cys94) were located in the mature peptide region. These eight conserved cysteines, including a characteristic adjacent cysteine pair (Cys53-Cys54), corresponded to the typical eight-cysteine motif of plant nsLTP proteins, suggesting that PgLTP possesses the conserved structural features of nsLTP family proteins.

The amino acid sequence, physiological and biochemical properties, tertiary structure, signal peptide, protein transmembrane structure, hydrophilicity/hydrophobicity, subcellular localization, and analytical phylogeny of the protein were predicted by determining the sequence of the *PgLTP* gene to reveal the characteristics of the LTPs. To identify potential cis-regulatory elements associated with *PgLTP* expression, the 5′-flanking sequence of PgLTP was analyzed using PlantCARE. Multiple regulatory elements were identified (see Supplementary Figure S3a online) , including a CAT-box (tissue-specific expression) at position +65, two STRE elements (stress response) at positions +34 and +40, and several TATA-box-like elements (core transcription). These results suggest that the 5′-flanking region of *PgLTP* contains potential cis-regulatory elements related to developmental and stress responses, providing preliminary insights into the possible regulation of *PgLTP* expression. PgLTP is a hydrophilic unstable protein (see Supplementary Figure S3b online) localized in the cell wall, with a theoretical isoelectric point of 8.36, indicating a positive charge at physiological pH conditions. PgLTP has a transmembrane structure (see Supplementary Figure S3c online) containing four α-helices (see Supplementary Figure S3d,e online) with no β-sheet structure, allowing it to efficiently transfer lip id molecules between cell membranes, thus playing an important role in intracellular lipid transport and metabolism. The AlphaFold2-predicted three-dimensional structure showed that the protein adopts a compact globular fold, consisting primarily of α-helices connected by loop regions (see Supplementary Figure S3d online). PgLTP possesses a signal peptide (see Supplementary Figure S3f online), which is positioned from the first to the twenty-second amino acid residues. The model displayed the canonical structural features of the plant non-specific lipid transfer protein (nsLTP) family, in which multiple α-helices form a stable hydrophobic cavity. Considering the abundance of conserved cysteine residues in the sequence, it is plausible that the protein maintains its structural integrity through intramolecular disulfide bonds. Moreover, the presence of a putative N-terminal signal peptide further supports its identity as a secretory LTP in plants. To construct the NJ phylogenetic trees, LTP sequences were BLASTed in the NCBI database, and the sequences with higher similarities were selected. As a result, the *pgLTP* gene sequence formed a distinct branch, with the protein sequence grouped with LTP from Diospyros lotus (XP_052175784), sharing only 62.7% sequence similarity. However, the bootstrap support value for this grouping was only 66% (see Supplementary Figure S4 online).

### 2.2. Construction and Validation of Transgenic Arabidopsis

**Figure 1:**
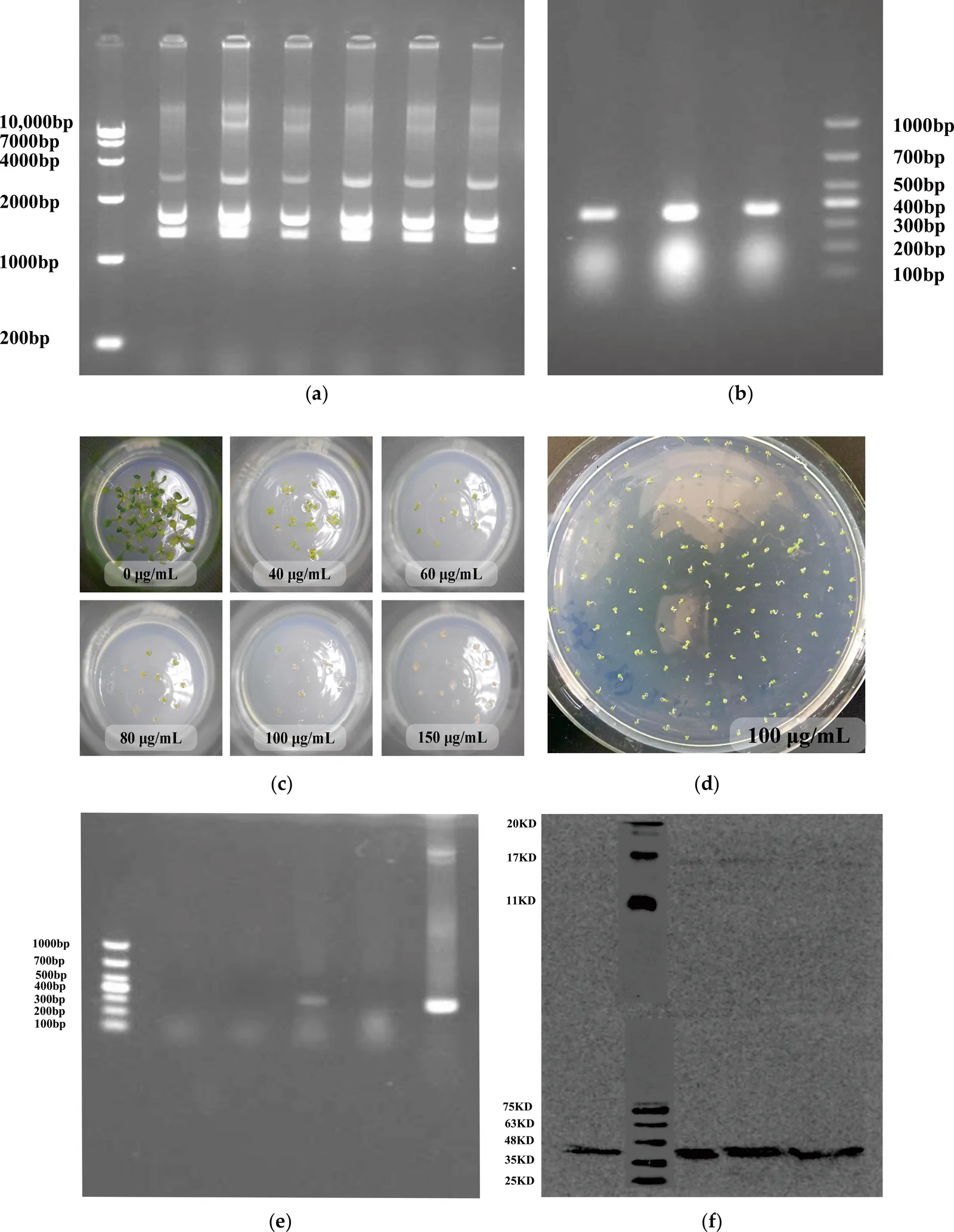
Transformation results and validation of transgenic *Arabidopsis*. (**a**–**c**) PCR results of pCAMBIA1303, DNA extracted from Agrobacterium tumefaciens, and DNA extracted from transformed Agrobacterium tumefaciens, respectively. (**d**) Western blot analysis of *PgLTP* expression in transgenic Arabidopsislines. Actin was used as the endogenous loading control to verify equal protein loading among samples. (**e**,**f**) Growth performance of wild-type and transgenic Arabidopsison hygromycin-containing medium. All experiments were performed with three independent biological replicates (*n*= 3).

In this study, transgenic Arabidopsis, which could express the *LTP* gene and translate into protein, was successfully constructed via *Agrobacterium tumefaciens*-mediated transfection. The pCAMBIA1303-*PgLTP* vector was successfully constructed using the cDNA reverse transcribed from RNA (Figure 1a) and transferred to Agrobacterium tumefaciens (Figure 1b).Wild-type *A. thaliana* could grow normally in the medium without hygromycin (Figure 1c), but growth inhibition was pronounced with an increase in hygromycin (Figure 1d). In the medium containing 40 μg/mL hygromycin, wild-type *A. thaliana* showed less effect on growth (Figure 1c); however, limited growth was observed in the medium containing 80 μg/mL hygromycin, with occasional green cotyledons (Figure 1c). Finally, it was unable to grow in the medium containing 100 μg/mL hygromycin (Figure 1c). Transgenic *A. thaliana* plants were able to grow and develop normally on medium containing 100 μg/mL hygromycin (Figure 1d), demonstrating successful expression of the hygromycin resistance gene.. Plants that exhibited normal growth were selected for subsequent validation to confirm the proper translation of the LTP gene into the PgLTP protein. The progeny of *A. thaliana*, which were transfected by a mixed Agrobacterium tumefaciens culture, expressed the LTP gene (Figure 1e) and translated it into protein (Figure 1f).

### 2.3. Antibacterial Activity Test

#### 2.3.1. Anti-Root Rot Activity Experiment

**Figure 2:**
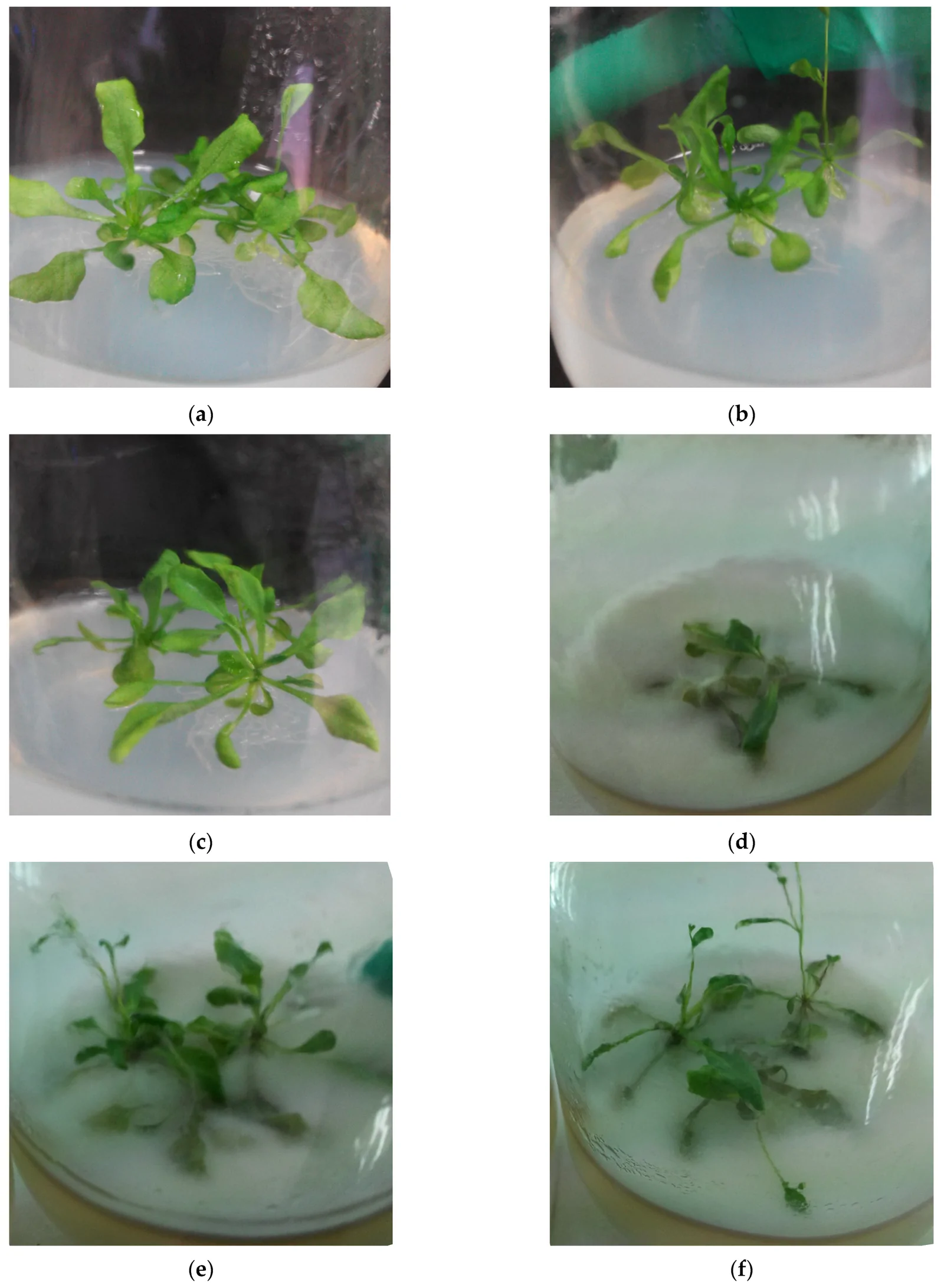

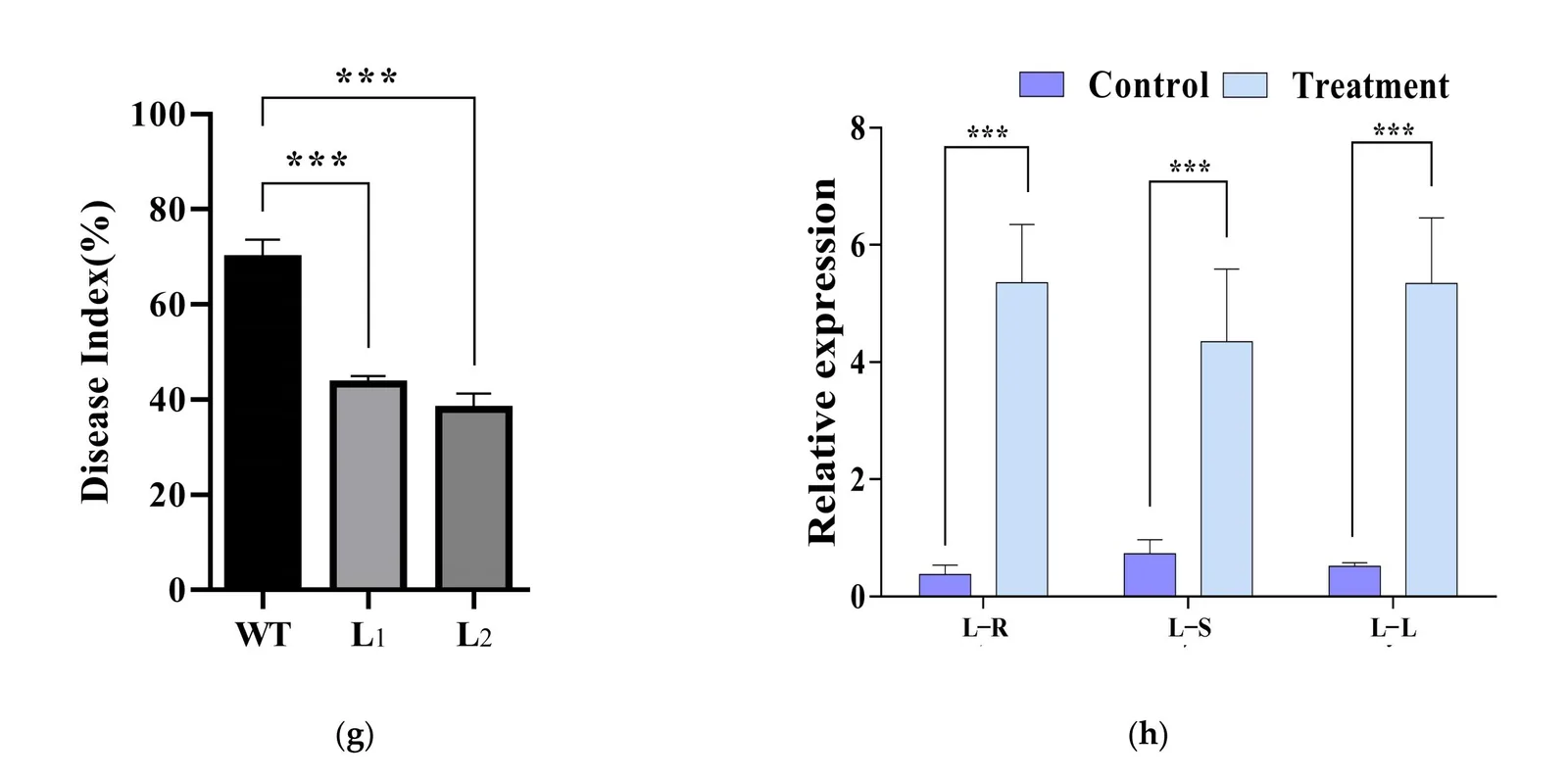
Phenotypic analysis of transgenic *Arabidopsis* upon *F. oxysporum* infection. (**a**–**c**) Non-inoculated control plants; (**d**–**f**) Plants inoculated with *F. oxysporum*. (**a**,**d**) WT; (**b**,**e**) transgenic line L_1_; (**c**,**f**) transgenic line L_2_. (**g**) Disease index of WT and transgenic lines after infection. (**h**) qRT-PCR analysis of *PgLTP* transcript levels in different tissues (R, root; S, stem; L, leaf) of transgenic lines L_1_ and L_2_. Values represent means ± SD (*n* = 3 biological replicates, ***p < 0.001).

Twenty-eight-day-old *A. thaliana* plants were inoculated with *Fusarium oxysporum*(*F. oxysporum*) spores (3.5 × 10^7^ spores/mL), and disease symptoms were evaluated after 7 days (Figure 2a–f). Compared with wild-type plants, *PgLTP*-overexpressing lines exhibited reduced leaf chlorosis, tissue decay, and root rot symptoms. The disease index of wild-type plants reached 69.58%, whereas the transgenic lines L_1_ and L_2_ showed significantly reduced disease indices of 43.21% and 37.97%, respectively (Figure 2g).

To determine whether differences in disease severity between transgenic *Arabidopsis* and wild-type plants were related to the expression of the LTP gene. The expression of LTP was detected by qPCR (Figure 2h). Healthy wild-type plants rarely expressed LTP after infection, but transgenic *Arabidopsis* expressed a large amount of LTP after infection. In summary, infection by *F. oxysporum* increases the expression of the LTP gene and helps *A. thaliana* to resist infection by *F. oxysporum*.

#### 2.3.2. Anti-Rust Fungus Activity

**Figure 3:**
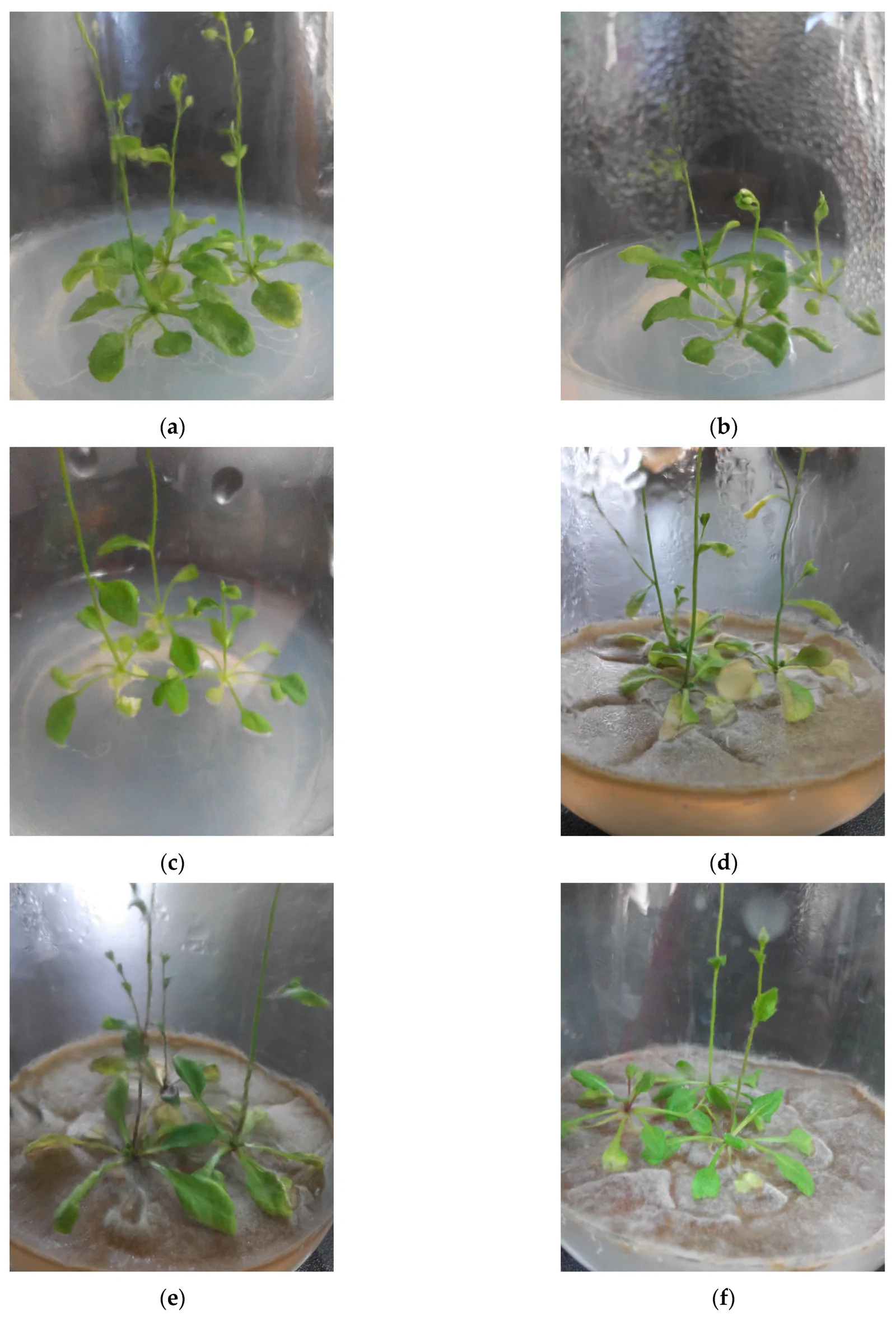

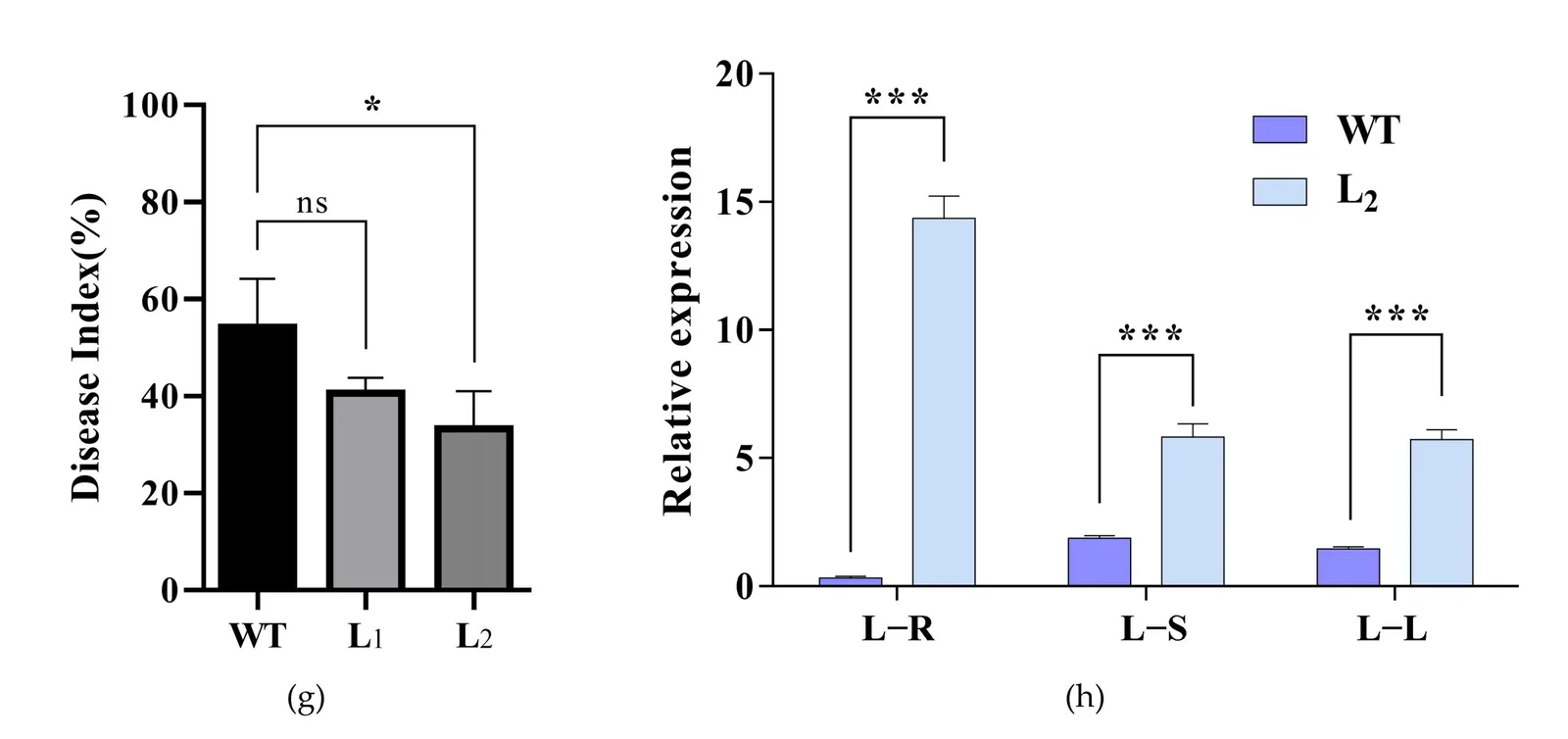
Phenotypic analysis of transgenic *Arabidopsis* upon *C. destructans* infection. (**a**–**c**) Non-inoculated control plants; (**d**–**f**) Plants inoculated with *C. destructans*. (**a**,**d**) WT; (**b**,**e**) transgenic line L_1_; (**c**,**f**) transgenic line L_2_. (**g**) Disease index of WT and transgenic lines after infection. (**h**) qRT-PCR analysis of *PgLTP* transcript levels in different tissues (R, root; S, stem; L, leaf) of transgenic lines L_1_ and L_2_. Data are shown as means ± SD (*n* = 3, ns, *p* > 0.05, * *p* < 0.05, *** *p* < 0.001).

Twenty-eight-day-old transgenic *Arabidopsis* was infected with 3.5 × 10^7^ spores/mL *Cylindrocarpon destructans* (*C. destructans*) for 6 days (Figure 3a–f). The wild-type rosettes were severely wilted and withered, and the stems and leaves were severely rotted, with 50% of the leaves withered. In contrast, transgenic *Arabidopsis* grew better, with no wilting, and the stems and leaves did not rot. After 7 days of inoculation, the disease index level of the wild type was concentrated at levels 2 and 3, with a disease index of 54.18%. In contrast, the disease index level of the transgenic *Arabidopsis* L_1_ was mostly at level 2, occasionally at level 3, with a disease index of 40.59%, while the disease index level of the L_2_ was mostly at level 2, occasionally at levels 1 and 3, with a disease index of 33.16% (Figure 3g).

To determine whether the different experimental results were related to LTP gene expression, the expression of LTP was detected by qPCR. The results revealed that transgenic *Arabidopsis* expressed 12-fold more LTP than the wild type after infection (Figure 3h).

The above experiments demonstrated that the expression of LTP is beneficial for the resistance of *A. thaliana* to infection by *F. oxysporum* and *C. destructans*.

### 2.4. Drought Stress Response

#### 2.4.1. Germination and Survival Rate Testing

The seeds were grown in 1/2MS medium containing mannitol, and the germination rates are shown in Supplementary Table S1 online. In the absence of mannitol, the germination rates of the transgenic and wild-type *Arabidopsis* were similar after day 4. After growth with 200 mM mannitol for 5 days, the germination rate of the wild-type *Arabidopsis* was only 63.0%, whereas the germination rate of transgenic *Arabidopsis* was 90.0%. Over time, the germination rate of the wild type was maintained at 78.0%, whereas the germination rate of transgenic *Arabidopsis* was 97.0%. When growing with 300 mM mannitol, the germination rate of transgenic *Arabidopsis* was still 90.0%, whereas that of wild-type *Arabidopsis* was only 63.0%.

The seedlings were subjected to drought stress until some plants died, after which they were re-watered. The survival rate of wild-type *Arabidopsis* was 80%, whereas that of transgenic *Arabidopsis* was 86–93%, indicating that LTP expression can effectively improve the survival rate of *Arabidopsis* seedlings under drought conditions.

#### 2.4.2. Physiological Parameter Assessment

**Figure 4:**
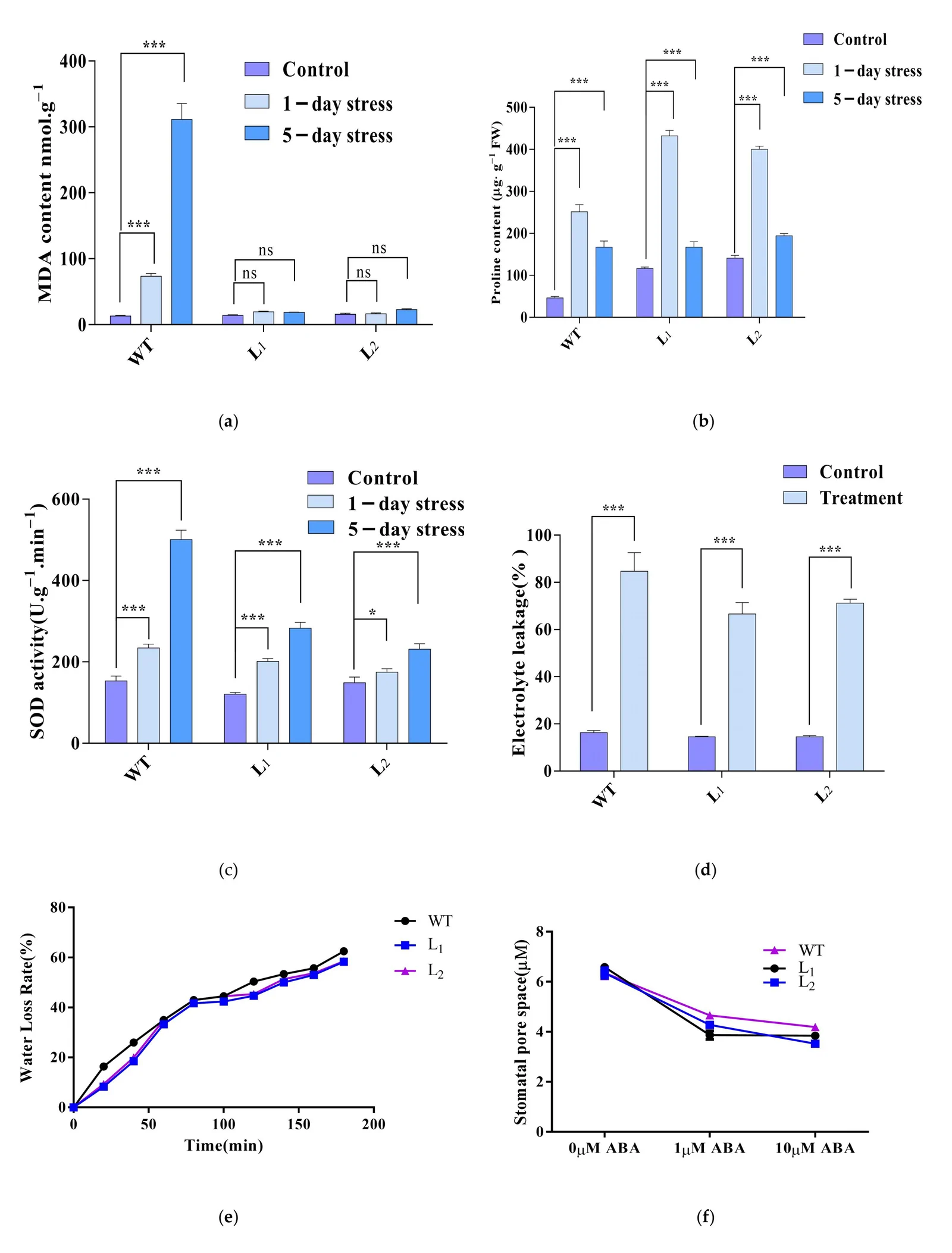

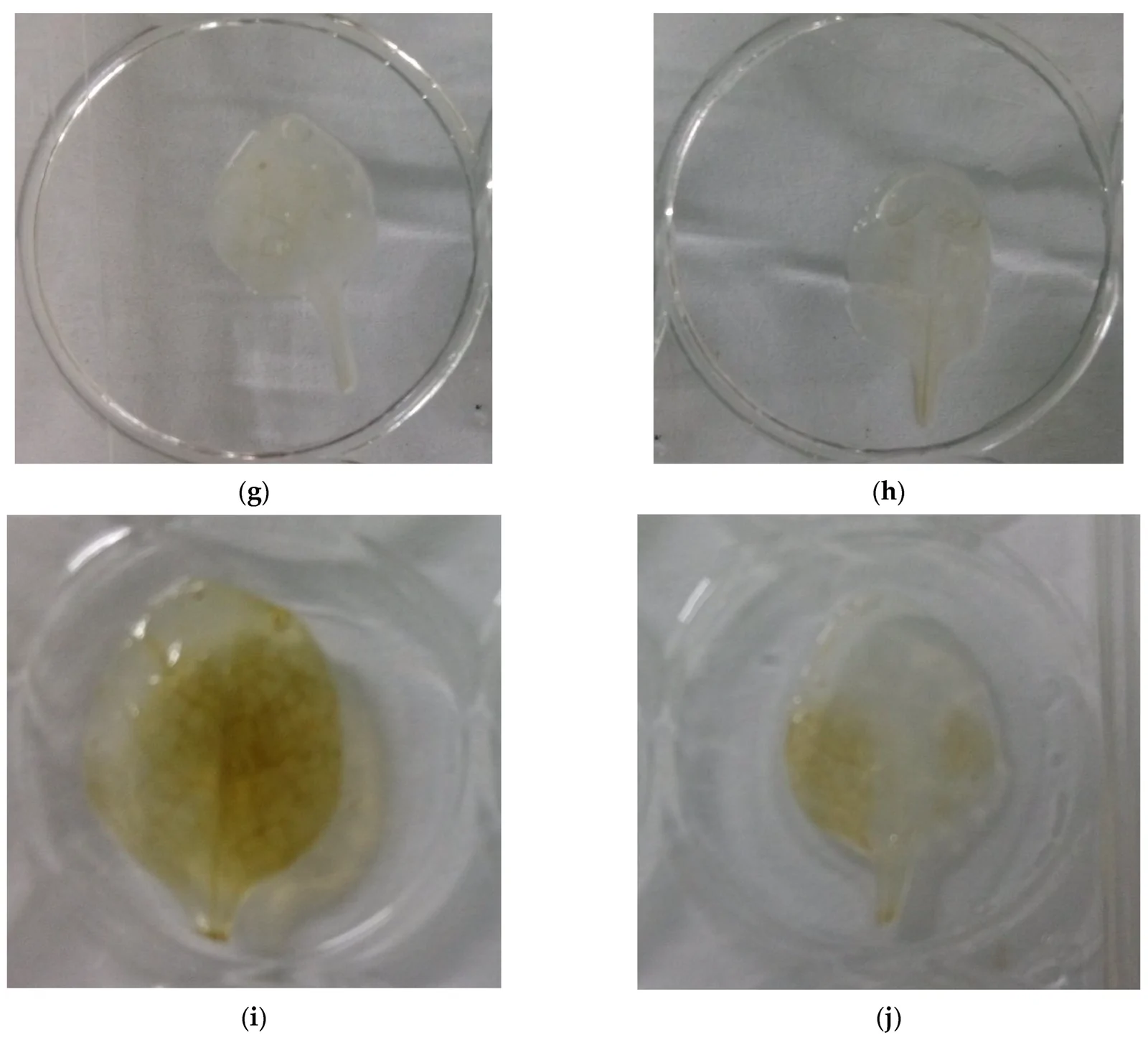
Detection of metabolites under drought stress. (**a**–**f**) Measurement of malondialdehyde (MDA) content, proline content, superoxide dismutase (SOD) activity, electrolyte leakage, water loss rate, and stomatal aperture, respectively; (**g**–**j**) DAB staining for detection of H_2_O_2_ accumulation: (**g**) WT plants before drought treatment; (**h**) transgenic plants before drought treatment; (**i**) WT plants after drought treatment; (**j**) transgenic plants after drought treatment. All data are presented as means ± SD (*n* = 3 biological replicates, ns, *p* > 0.05, * *p* < 0.05, *** *p* < 0.001).

To determine how LTP enhances drought resistance in transgenic *Arabidopsis*, we measured the content of malondialdehyde, proline, and SOD (Figure 4). Under stress for 5 days, the malondialdehyde content increased to 316.24 nmol·g^−1^ in wild-type plants, but only to 19.6 nmol·g^−1^ and 21.66 nmol·g^−1^ in transgenic *Arabidopsis*. The SOD content of wild-type plants increased to 499.88 U·g^−1^·min^−1^, while that of two transgenic *Arabidopsis* plants increased to 277.88 U·g^−1^·min^−1^ and 224.22 U·g^−1^·min^−1^, respectively. LTP gene expression significantly reduced the increased malondialdehyde and SOD contents in transgenic *Arabidopsis* relative to the wild type, but increased the proline content. Under stress for 1 day, the proline content showed 1.55- and 1.46-fold increases in transgenic *Arabidopsis* compared to wild-type plants, and by day 5, the proline content decreased to normal levels in both transgenic *Arabidopsis* and wild type.

At the 3 h time-point, the water loss rates from the rosette leaves of wild-type *Arabidopsis*, L_1_, and L_2_ were about 62.52%, 58.33%, and 58.52%, respectively (Figure 4e). Within the first 50 min, transgenic *Arabidopsis* exhibited a lower water loss rate than the wild type. These results suggested that overexpression of LTP in *A. thaliana* enhanced resistance to water loss.

As shown in Figure 4g,h, the leaves of both wild-type and transgenic *Arabidopsis*, which were stained with DBA, turned yellow following a 5-day period of drought stress. This observation suggested that water deficit induced physiological damage to the leaves, likely associated with the accumulation of H_2_O_2_. As drought conditions persisted, the degree of yellowing intensified. In transgenic plants, discoloration was confined to specific localized regions, whereas wild-type plants displayed widespread yellowing across the entire leaf surface. These findings indicated that drought-induced oxidative stress contributed to leaf damage, and that *PgLTP* exhibited reduced H_2_O_2_ accumulation under drought stress, suggesting that *PgLTP* may contribute to maintaining cellular redox balance during stress.

Under normal conditions, there was no significant difference in the electrolyte concentration between transgenic and wild-type *Arabidopsis* (Figure 4d). After 14 days of drought treatment, the electrolyte concentration in wild-type *Arabidopsis* was 84%, while that in the different lines of transgenic *Arabidopsis* was 66% and 71%, significantly lower than that of wild-type *Arabidopsis*. This suggested that overexpression of *PgLTP* reduced the electrolyte concentration in *Arabidopsis*, suggesting improved membrane stability under salt stress and enhancing the salt tolerance of transgenic *Arabidopsis*.

As shown in Figure 4f, prior to treatment, the stomatal lengths of wild-type and transgenic *Arabidopsis* were essentially the same. Following treatment with 1 μM abscisic acid (ABA), the stomatal length was reduced in both lines; however, the transgenic plants showed a more rapid decrease, resulting in shorter stomata. With 10 μM ABA treatment, a similar trend was observed, although the stomatal length of the L_1_ line no longer decreased noticeably. These results suggested that LTP may enhance stomatal closure in *Arabidopsis* and facilitate a more rapid response to ABA-associated responses.

### 2.5. Response to High Salt Stress

**Figure 5:**
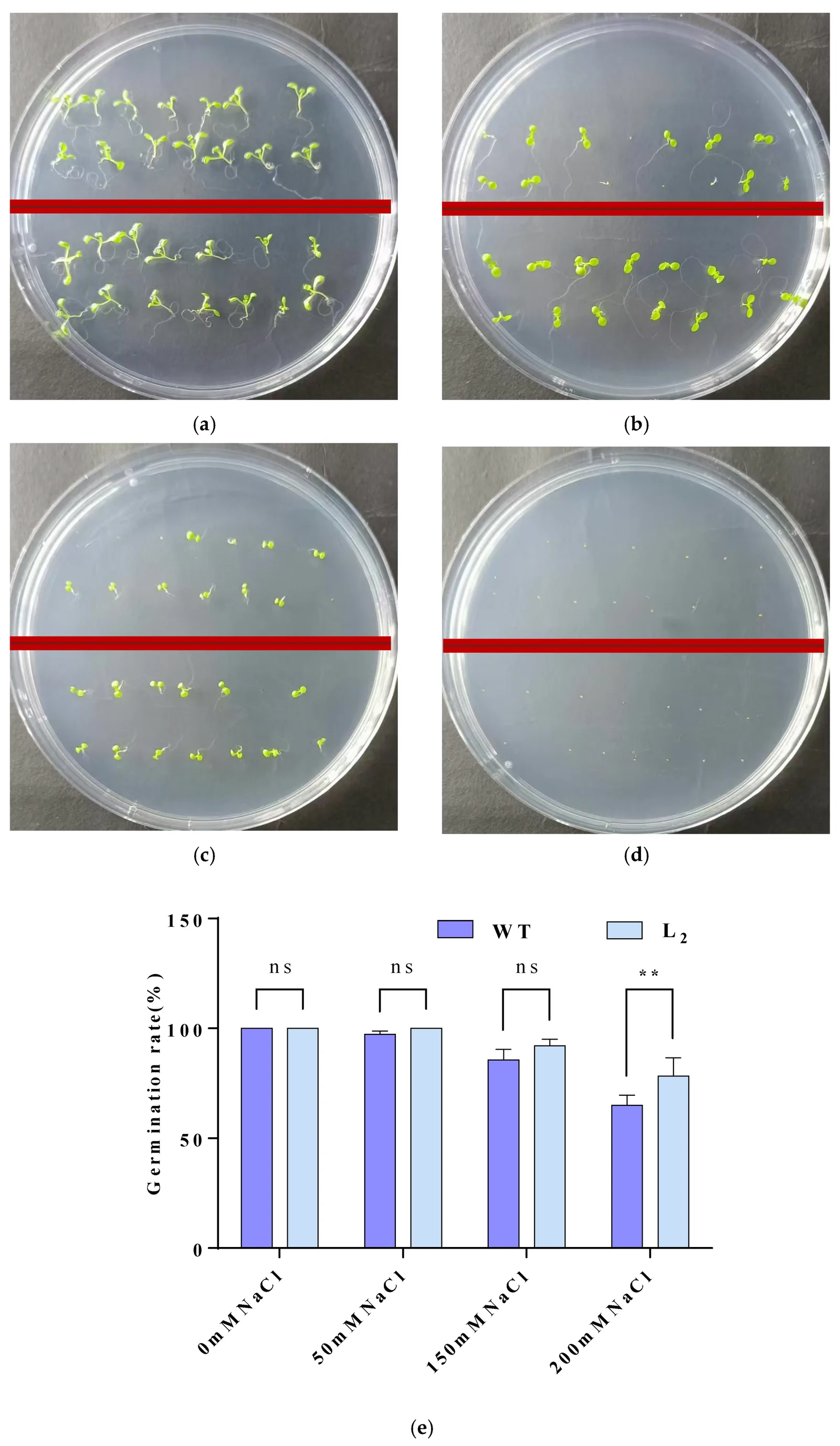
Germination rate of *Arabidopsis* seeds under high salt stress. (**a**–**d**) Germination of WT (**upper panels**) and transgenic lines (**lower panels**) on medium supplemented with 0, 50, 150, and 200 mM NaCl, respectively. (**e**) Quantification of germination rates. Data are shown as means ± SD (*n* = 3 three independent experiment, ns,* p* > 0.05, ** *p* < 0.01, with at least 50 seeds per replicate).

Seeds of *A. thaliana* were sown on 1/2 MS medium for 7 days, and the germination rate of the seeds was calculated (Figure 5a–e). When grown on 1/2 MS medium with 150 mM NaCl, the germination rate of transgenic *A. thaliana* L_1_ and L_2_ was slightly higher than that of wild-type *A. thaliana*, and this difference increased with the concentration of NaCl; i.e., the LTPs were able to help *A. thaliana* improve its resistance to drought stress.

**Figure 6:**
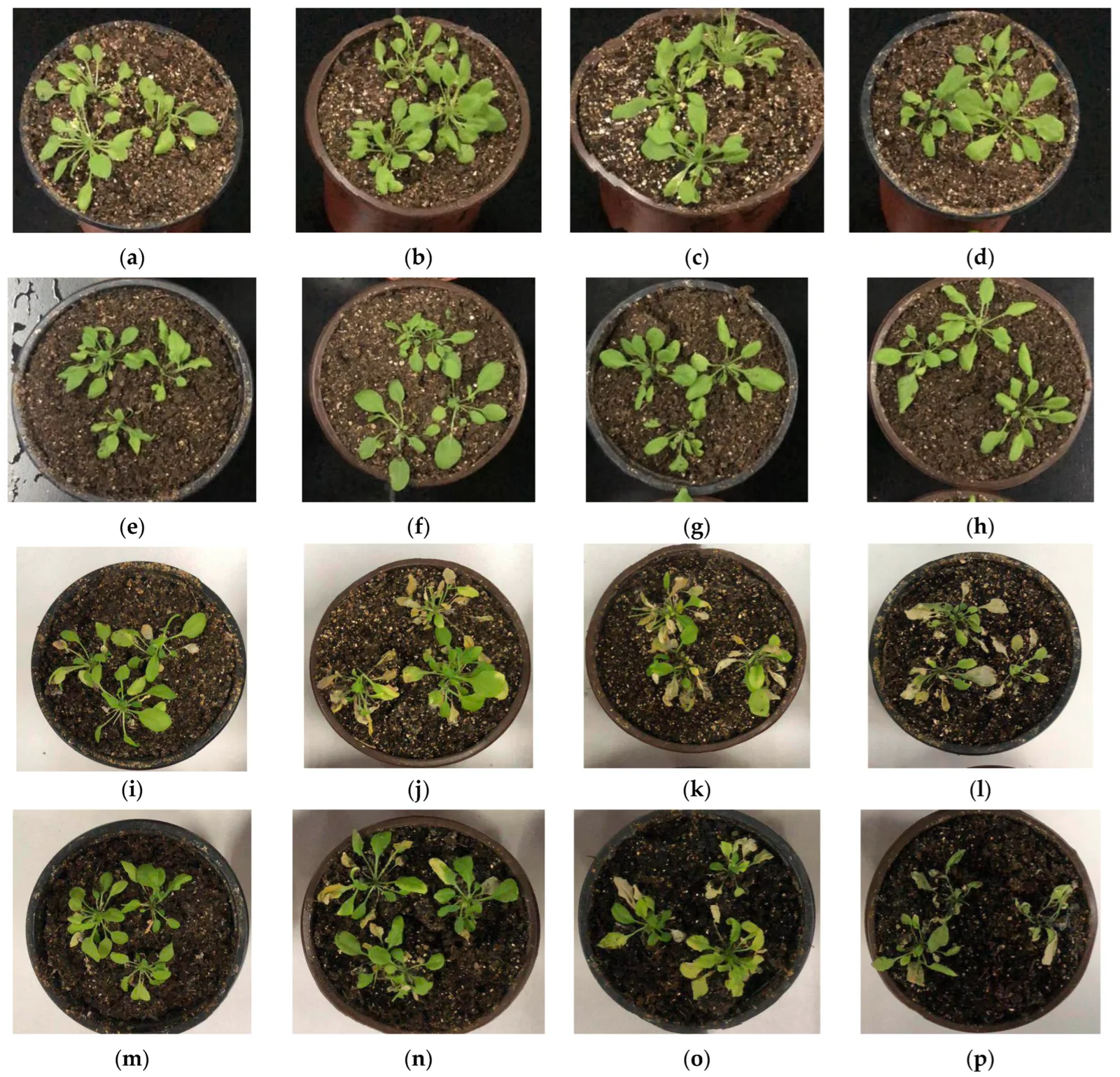
Growth of *A. thaliana* seedlings with salt stress. (**a**–**d**) WT plants before treatment; (**e**–**h**) Transgenic lines before treatment. (**i**,**m**) 0 mM NaCl; (**j**,**n**) 100 mM NaCl; (**k**,**o**) 200 mM NaCl; (**l**,**p**) 300 mM NaCl. Photographs were taken 10 days after treatment. Experiments were repeated three times with similar results (*n* = 3 biological replicates).

When seedlings were subjected to high salt stress (Figure 6a–p), the transgenic *A. thaliana* showed similar growth to wild-type *A. thaliana* before stress (Figure 6a–h); however, after rehydration, the transgenic and wild-type *A. thaliana* began to show phenological differences under 100 mM NaCl (Figure 6j,n), and the survival rate difference between transgenic and wild-type *A. thaliana* increased with NaCl concentration, which indicated that the LTPs can increase plant resistance to salt stress.

**Figure 7:**
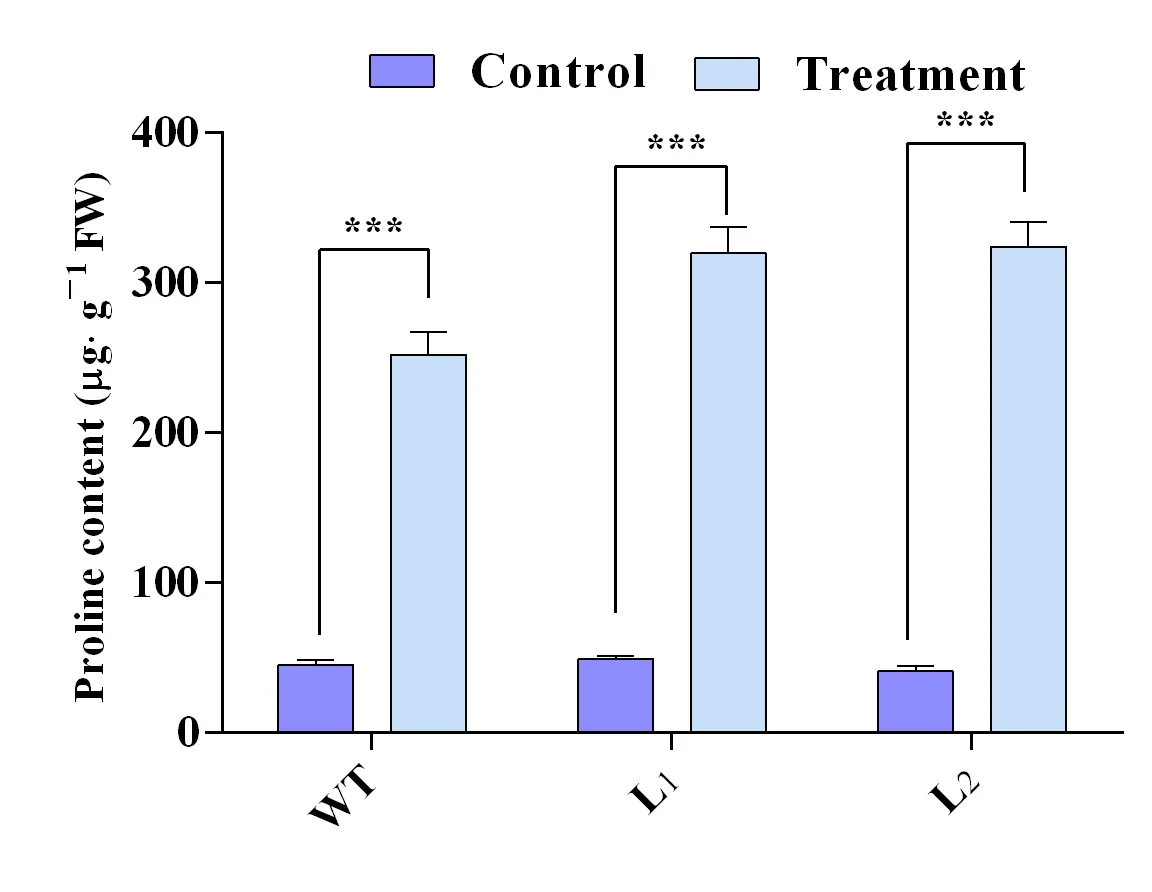
Proline content of WT and transgenic *Arabidopsis* under salt stress. Proline accumulation was measured after NaCl treatment. Data are presented as means ± SD (*n* = 3 biological replicates, ns, *p* > 0.05, *** *p* < 0.001,).

We next determined the proline content of transgenic *Arabidopsis* to establish how LTP enhances salt tolerance in transgenic *Arabidopsis* (Figure 7). The proline content significantly increased after stress, with a larger increase in transgenic *Arabidopsis* compared to wild-type *Arabidopsis*, with wild-type *Arabidopsis* showing an increase of 193.34 after stress, whereas transgenic *Arabidopsis* showed a minimum increase of 260.3.

## 3. Discussion

In plant LTPs, the quantity and spatial arrangement of cysteine residues have pivotal roles in determining structural integrity and functional diversity. Canonical LTPs typically contain eight conserved cysteine residues (C-Xn-C-Xn-CC-Xn-CXC-Xn-C-Xn-C) that form four disulfide bonds, which are essential for maintaining protein stability [13]. Intriguingly, our study revealed that *PgLTP* has ten cysteine residues, a characteristic shared with atypical LTPs. This distinctive feature suggested the potential formation of additional disulfide bonds that may enhance structural rigidity under stress conditions. The additional cysteine residues may contribute to structural diversity of PgLTP; however, their specific functional roles require further investigation [7]. Cysteine residues are fundamental for forming disulfide bonds, which are critical for maintaining protein structure and functionality [14,15]. The presence of additional cysteine residues may contribute to the structural diversity of plant LTPs and potentially influence their functional properties [16]. While conventional LTPs containing eight cysteine residues form four disulfide bonds [17] and typically exhibit molecular weights between 6.5 and 10.5 kDa [18], the LTP characterized in this study displayed features: ten cysteine residues capable of potentially contributing to disulfide bond formation, a molecular weight of 12 kDa, four α-helices, a transmembrane domain, a signal peptide, and an elevated isoelectric point (pI = 8.36). These characteristics indicate that *PgLTP* possesses distinct structural features compared with previously characterized plant LTPs [9,10].

To ensure that the classification of the LTP in this study was comparable to that reported for other LTPs, we constructed phylogenetic trees for analysis. The BLASTn result of the LTP DNA sequence showed that no similar sequence was reported; therefore, sequences of LTP from other species were collected to construct a phylogenetic tree. The phylogenetic tree of the DNA sequence showed that LTP is a separate branch existing on its own. The phylogenetic tree of protein sequences showed that LTP is grouped with LTP of Diospyros lotus (XP_052175784); however, as the bootstrap support value is only 66%, it cannot be completely identified as the same species [19,20,21]. Compared to traditional plant LTP classification methods [22,23] and the LTP classification method of Boutrot [24], the LTP isolated from *Panax ginseng* represents a member of the plant *nsLTP* family with distinct structural characteristics. Moreover, the impact of its ten cysteine residues on functionality and the functional differences compared to other LTPs must be validated in subsequent experiments.

Transgenic *Arabidopsis* showed enhanced resistance to infection with *F. oxysporum* and *C. destructans*. PgLTP transcript and protein accumulation were detected in transgenic lines, whereas they were not detected in wild-type plants [25]. Some studies have shown that LTP protein can reduce damage by inhibiting the growth of *F. oxysporum* [16]. Previous studies have suggested that plant LTPs are involved in biotic stress responses through their roles in lipid transport, membrane-associated processes, and interactions with defense-related pathways [26,27], thus increasing resistance to biotic stress. Compared with traditional plant LTP classification methods [22,23] and the classification criteria proposed by Boutrot et al. [24], *PgLTP* was classified as a member of the plant *nsLTP* family and displayed distinctive cysteine distribution characteristics. The contribution of these cysteine residues to PgLTP structure and function remains to be further investigated.

In this study, LTP greatly improved *A. thaliana* seed germination and seedling survival under both drought and salt stress conditions, with differences in metabolites detected during metabolic reactions. Related investigations have shown that LTP can be synthesized in large quantities in the whole plant under abiotic stress [11], increasing the resistance of the whole plant to abiotic stress. However, previous studies have also reported that LTP expression patterns may vary among plant species and stress conditions, suggesting diverse roles of LTPs in abiotic stress responses [28,29]. The ability of the LTP gene to improve plant resistance to abiotic stresses, such as drought and salt stress, has been reported [30,31,32]. In this study, transgenic *Arabidopsis* improved tolerance to drought stress, mainly by increasing proline synthesis and decreasing malondialdehyde and SOD contents. A large reduction in malondialdehyde implies a large reduction in oxidative damage and a significant increase in the antioxidant capacity of the plant, while LTP gene expression also improves the antioxidant capacity of the plant [33]. At the same time, transgenic *Arabidopsis* increased the proline content, which alters the intracellular osmotic potential and improves water retention and uptake abilities [34,35], thus enhancing the drought tolerance of the plant. In salt tolerance experiments, we examined the difference in proline synthesis between transgenic and wild-type *A. thaliana* under high salt conditions, and the results indicated that LTP enhances plant tolerance to high salt by increasing proline synthesis. Previous studies have suggested that some LTP members may be involved in defense-related processes, including interactions with salicylic acid-, jasmonic acid-, and flavonoid-associated pathways [31,36].

Based on the results of transgenic *Arabidopsis* experiments under salt and drought stress, this study further demonstrated that PgLTP contributes to plant stress tolerance through associated physiological responses. Under drought conditions, the transgenic *Arabidopsis* displayed a notably improved water retention capacity, with a 4.19% higher retention rate than that of the wild-type plants after 3 h of stress treatment, particularly within the first 50 min of exposure. This is consistent with previous findings in which the heterologous expression of LTP in tomato also reduced water loss. In tomato, LTP enhanced cuticular wax deposition and promoted stomatal closure to minimize water loss [37]; similarly, in this study, stomata of the transgenic *Arabidopsis* responded rapidly to ABA, thus reducing water loss.

Moreover, PgLTP overexpression was associated with reduced ROS accumulation under stress conditions. After five days of stress, the transgenic *Arabidopsis* exhibited only partial yellowing of the leaves, whereas the wild-type plants showed complete leaf yellowing. This difference may be attributed to LTP overexpression, which effectively and rapidly reduces ROS levels. Previous studies have indicated that specific LTP members may influence ROS accumulation, suggesting that LTPs may be involved in oxidative stress responses [38]. In that study, ROS bursts led to extensive cell death and substantial electrolyte leakage. In contrast, in the present study, PgLTP overexpression was associated with reduced ROS accumulation and decreased electrolyte leakage by 18% compared with wild-type Arabidopsis. The lower electrolyte leakage observed in PgLTP-overexpressing plants suggests improved membrane stability under stress conditions [38,39].

Taken together, PgLTP overexpression was associated with improved stress-related physiological responses, including altered ROS accumulation, stomatal responses, membrane stability, and proline accumulation.

The different responses demonstrated by LTP in studies of abiotic stress resistance may be explained by promoter differences [40] or different SNPs [41]. As the LTP isolated in this study is structurally distinct from common LTPs, its functional differences from other LTPs and metabolic differences with respect to abiotic stresses need to be investigated and verified further.

## 4. Materials and Methods

### 4.1. Materials

Three-year-old ginseng plants were collected from Beigang Town, Fusong County, Jilin Province, and identified by the Department of Traditional Chinese Medicine Identification of Changchun University of Traditional Chinese Medicine. Competent cells (*E. coli DH5α*) were purchased from Shanghai Biotechnology; the vector pCAMBIA1303 with a 35S strong promoter and nopaline synthase transcriptional terminator was purchased from Beijing Dingguo Changsheng Biotechnology Co., Ltd., Beijing, China; and the Columbia-0 seeds (Col-0) of *A. thaliana* were provided by the Bioengineering Laboratory of Changchun University of Chinese Medicine. *F. oxysporum* and *C. destructans*, isolated from *Panax ginseng*, were obtained from Prof. Yang Limin, who worked at the State Key Laboratory of Ecological Recovery and Ecosystem Management.

### 4.2. Acquisition of Target Gene and Protein Sequences

Based on the ginseng transcriptome data from the National Center for Biotechnology Information (Accession: AMX81288), primer sequences were designed using Primer 5 and synthesized by Huada Genetics (Beijing, China). The primer sequences and PCR program are shown in the Supplemental Data.

*PgLTP* sequences from other species, together with the sequence obtained in this study, were used to construct a neighbor-joining phylogenetic tree with default parameters using MEGA X v10.0.5.

### 4.3. Bioinformatic Analysis of PgLTP

To predict potential cis-regulatory elements within the 5′ flanking region of PgLTP, PlantCARE analysis was performed. The 2 kb upstream of the transcriptional start site (AAA) of the LTP genes was selected and considered as the gene promoter sequence. The cis-acting elements of the LTP promoter were predicted using the PlantCARE online website (https://bioinformatics.psb.ugent.be/webtools/plantcare/html/accessed on 30 July 2026) and visualized with TBtools v1.098.

The basic physicochemical characteristics of proteins were analyzed using Expasy-ProtParam (https://web.expasy.org/protparam/accessed on 30 July 2026). Multiple sequence alignment and cysteine conservation analysis. The deduced amino acid sequences of LTPs were aligned using MAFFT v7 with default parameters. Signal peptides were predicted by SignalP 5.0 and removed prior to alignment. The resulting alignment was visualized and edited using ESPript 3 (https://espript.ibcp.fr accessed on 30 June 2026), with conserved cysteine residues highlighted. Sequence identity percentages were calculated based on pairwise comparisons. The basic physicochemical characteristics of proteins were analyzed using Expasy-ProtParam, and hydrophilicity/hydrophobicity was evaluated using ExPASy-ProtScale (https://www.expasy.org/resources/protscale accessed on 30 April 2026). Protein signal peptides were analyzed using SignalP-5.0, and protein transmembrane structures were analyzed using DeepTMHMM (https://dtu.biolib.com/DeepTMHMM accessed on 30 April 2026). Protein secondary structure was analyzed using Phyre2 (https://www.sbg.bio.ic.ac.uk/phyre2 accessed on 30 July 2026), and the tertiary structure was predicted using AlphaFold2 via the ColabFold platform (https://colab.research.google.com/github/sokrypton/ColabFold/blob/main/AlphaFold2.ipynb accessed on 30 July 2026). Subcellular localization was analyzed using Cell-PLoc 2.0.

### 4.4. Construction and Validation of Transgenic A. thaliana

The total RNA of ginseng was extracted using a modified TRIzol method [42] and then amplified and reverse transcribed using TaKaRa’s PrimeScript RT reagent kit. The product of reverse transcription was ligated to the pCAMBIA1303 vector. The successfully ligated product was then transferred into E. coli *DH5α* cells and cultured on a KanR-containing medium (30 μg/mL) for selection. Successfully transformed receptor cells were cultured in KanR-containing medium, and plasmids were extracted. Agrobacterium-mediated transfection experiments were performed according to a previously described method [43].

Wild-type *Arabidopsis* seeds were sequentially surface-sterilized by soaking in 75% ethanol for 5 min, rinsing with sterile water twice, soaking in 2% sodium hypochlorite for 5 min, and rinsing with sterile water 3–5 times. Then, the seeds were dried with aseptic absorbent paper, uniformly sown in 1/2 MS medium containing hygromycin at different concentrations (0, 40, 60, 80, 100, 150 μg/mL), and then sealed and incubated at 22 °C. The growth of the seeds was observed daily.

The surface-sterilized transgenic *Arabidopsis* seeds were sown in 1/2 MS medium containing hygromycin, which was based on the results of the abovementioned experiment, and then sealed and cultured under 16 h of light/8 h of darkness at 21–23 °C.

Total RNA was extracted from normally growing transgenic *Arabidopsis* seedlings and reverse-transcribed according to the method described in Section 4.2 to evaluate the transcriptional expression of the target gene. The expression levels of the target gene were normalized using β-Actin as the endogenous reference gene, and the primer sequences used for qRT-PCR analysis are listed in Table S3. Total proteins were subsequently extracted from the same materials, and Western blot analysis was performed to evaluate the translational expression of the target gene. Protein loading was normalized using Plant-Actin as the endogenous reference protein. Actin expression was detected using an Anti-Plant Actin Mouse Monoclonal Antibody (3T3) (Abbkine, Catalog No. A01050) as the loading control, and PgLTP protein accumulation was analyzed by Western blot.

### 4.5. Biotic Stress Resistance of Transgenic Arabidopsis

#### Detection of Plant Responses to Biotic Stress

Surface-sterilized transgenic seeds were cultured in 1/2 MS medium for 28 days, and seedlings of uniform growth size were selected. Subsequently, 1 mL of the spore suspension was added dropwise around the edge of the medium in a triangular bottle, which was then tilted at an angle of 45° and shaken to evenly distribute the spore suspension on the surface. After sealing, the seedlings were placed in a dark environment with 95% humidity at 22 °C for 24 h and then transferred to the conventional growth environment to check the disease status every 24 h, and the disease index was calculated after 7 days of infection.

The disease severity of *F. oxysporum* infection was evaluated according to a previously described scoring system [44]. Disease symptoms were classified into five levels based on the degree of leaf chlorosis and tissue decay: level 0, no visible symptoms; level 1, chlorosis at the inoculation site accompanied by a hypersensitive necrosis reaction; level 2, less than 50% of the leaf area showing rot symptoms; level 3, 50–75% of the leaf area showing rot symptoms; and level 4, complete rotting and whitening of leaves and petioles. To minimize observer bias, disease severity was independently evaluated by two researchers who were blinded to the genotype information, and the average score was used for subsequent analysis. The disease index was calculated using the following formula:

disease index = ∑number of leaves in each level × number of levels/(total number of leaves × number of highest level) × 100%.

Before adding the spore suspension, the *Arabidopsis* leaves were cut, wrapped in tin foil, and quickly placed in liquid nitrogen for brief storage before total RNA extraction. Similarly, photographs were taken after 7 days of treatment and samples were collected and stored briefly in liquid nitrogen before total RNA extraction. The extracted total RNA was reverse transcribed according to the method described in Section 4.4, and the reverse transcription products were quantitatively analyzed by qPCR. The details of the qPCR primers and amplification protocol are shown in Tables S2 and S3.

### 4.6. Drought Tolerance Experiment of Transgenic Arabidopsis

#### 4.6.1. Germination Rate and Survival Rate Testing

Germination rate: Surface-sterilized *Arabidopsis* seeds were sown in 1/2 MS medium containing 0, 200, 300, or 400 mM mannitol, vernalized for 3 days at 4 °C, and then cultivated under controlled conditions at 22 °C, with a photoperiod of 16 h of light followed by 8 h of dark. Seeds with germination lengths ≥ 1 mm were counted daily.

Seedling experiment: Four-week-old *A. thaliana* plants were watered, and then no further watering was applied. When some plants died because of water deprivation, re-watering was initiated, and the survival rate was counted after 7 days. Three replicates were set up for each line, with ten plants per replicate.

#### 4.6.2. Physiological Parameter Assessment

Water loss rate: Plants were grown for 4 weeks, and rosette leaves were collected. Under constant temperature (25 ± 1 °C) and humidity (60%), the samples were weighed every 20 min for 180 min. The average water loss rate of the rosette leaves was calculated based on the fresh mass basis. This analysis was performed with three replicates, each with three plants.

Determination of ROS: Four-week-old *A. thaliana* plants were watered, and then no further watering was applied for 7 days. The leaves were examined by diaminobenzidine (DAB) staining to determine the H_2_O_2_ accumulation, according to a previously described method [45]. The experiment was repeated three times using ten leaves for each line.

Determination of electrolyte leakage: Four-week-old *A. thaliana* was allowed to fully absorb water, and then no more water was administered for 14 days. The leaves were collected, and 0.30 g of wild-type and positive *A. thaliana* leaves (undamaged) before and after the forced conditions were placed in a test tube with 20.0 mL of deionized water. The tube was covered and shaken at 150 rpm at room temperature for 16 h, then set aside. Next, the temperature was stabilized to around 20 °C, and the contents were rinsed thoroughly with deionized water. The electrolyte conductivity was measured (C1). Then, the leaves were sterilized for 10 min at 121 °C to ensure the full release of the electrolytes. After cooling to room temperature, the electrolyte conductivity was measured again (C2). The electrolyte rate was calculated as L = C1/C2. In each experiment, three samples were obtained from three plants, and the experiment was repeated three times.

Fourteen-day-old *Arabidopsis* leaves were used to prepare lower epidermal peels, which were then incubated in a HEPES buffer and subjected to continuous high-intensity light for 12 h to promote full stomatal opening. Afterwards, the samples were transferred into HEPES solutions supplemented with either 1 µM or 10 µM ABA. Following a 3 h treatment, stomatal apertures were examined and quantitatively measured under a light microscope.

### 4.7. Salt Resistance Experiment of Transgenic Arabidopsis thaliana

Germination rate: Surface-sterilized seeds were cultured in 1/2 MS medium containing 0, 50, 150, or 200 mM NaCl, vernalized for 3 days at 4 °C and then placed in an artificial climate box for light culture. We counted the number of germinated seeds with germination lengths ≥ 1 mm each day.

Four-week-old *A. thaliana* were subjected to salt stress treatment by applying NaCl solutions at concentrations of 0, 100, 200, and 300 mmol/L. The plants were watered with the solutions every three days for a total of three treatments. After salt treatment, the plants were watered normally, and the condition of the plants was observed after 7 days.

### 4.8. Determination of Enzyme Activity and Metabolic Substances

The content of malondialdehyde and proline was measured according to methods previously reported in the literature [46,47]. After grinding the mixture containing 0.5 g of tissue and 2.5 mL of phosphate-buffered saline (PBS) into a homogeneous slurry, 2.5 mL of PBS was added and mixed thoroughly. The homogenate was then transferred to a centrifuge tube and centrifuged at 10,000 rpm for 15 min at 4 °C. A portion of the supernatant was taken and diluted appropriately, and the optical density of the reaction solution was measured at 560 nm using the nitrogen blue tetrazolium (NBT) photochemical reduction method. Data collected from the measurements were used to calculate the superoxide dismutase (SOD) activity.

### 4.9. Statistical Analysis

Results are presented as the mean ± SD. Each experiment was repeated at least three times to ensure reproducibility. Statistical significance was determined using one-way analysis of variance with Tukey’s multiple comparison test or an unpaired two-tailed Student’s t test. A *p* value of less than 0.05 was considered statistically significant, and *p* < 0.01 and *p* < 0.001 indicated highly and very highly significant differences, respectively.

## 5. Conclusions

Based on the results of transgenic *Arabidopsis* experiments under salt and drought stress, this study demonstrated that PgLTP contributes to plant stress tolerance. Previous studies have suggested that some LTP members may participate in defense-related processes associated with salicylic acid-, jasmonic acid-, and flavonoid-related pathways. In this study, PgLTP exhibited reduced ROS accumulation and improved membrane stability under stress conditions, suggesting that PgLTP may contribute to oxidative stress adaptation. However, the detailed molecular mechanisms underlying PgLTP-mediated stress responses require further investigation.

## Data Availability

The original contributions presented in this study are included in the article and Supplementary Materials. Further inquiries can be directed to the corresponding authors.

## Supplementary Materials

The following supporting information can be downloaded at: https://www.mdpi.com/article/10.3390/ijms27167094/s1.
