# Supplementary material for: Bioinformatic Characterization and Functional Analysis of a Lipid Transfer Protein from Panax ginseng Involved in Biotic and Abiotic Stress Responses
Source: Int J Mol Sci. 2026 Aug 7;27(16):7094. doi: 10.3390/ijms27167094 (PMC13513311; doi:10.3390/ijms27167094)
Supplement: Supplementary file 1 [file ijms-27-07094-s001.zip › ijms-4437924-supplementary.pdf]

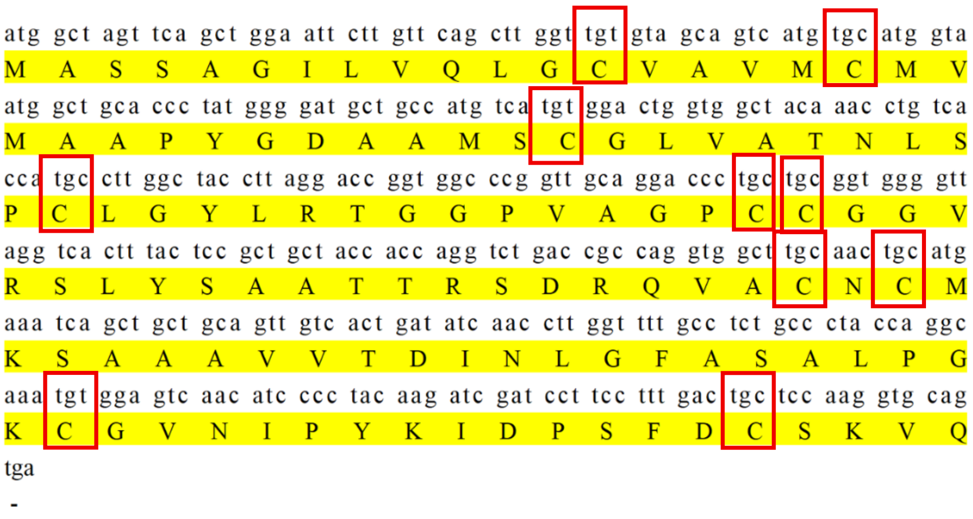

Figure S1. Analysis of *PgLTP* sequences.

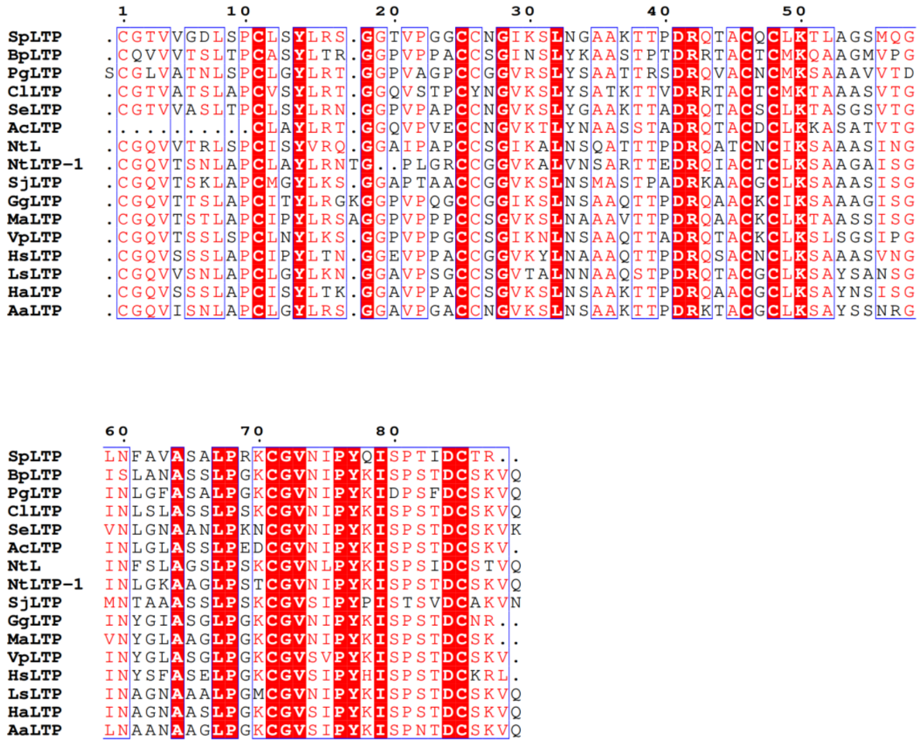

Figure S2. Multiple sequence alignment of *PgLTP* with representative plant *nsLTPs*. The conserved cysteine residues across all sequences are highlighted in red.

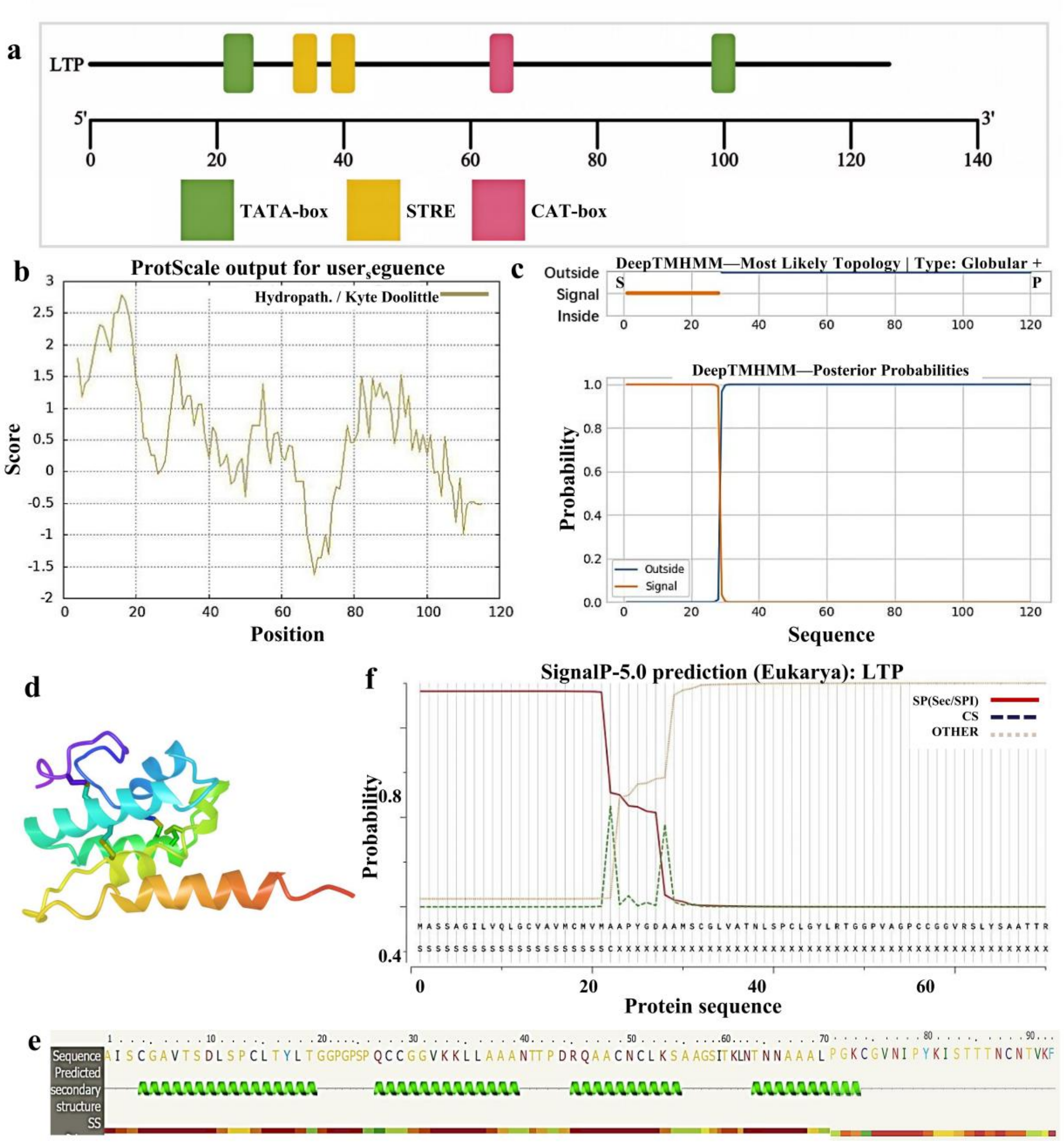

a: Cis-acting element analysis of the promoter region of *LTP* genes. The distribution of cis-acting elements in *LTP* promoters. Blocks with different colors represent various types of cis-acting elements; b: Analysis results of hydrophilic/hydrophobicity; c: Analysis results of transmembrane structure; d: Analysis results of three-dimensional structure; e: Analysis results of secondary structure; f: Analysis results of signal peptide.

Figure S3. Bioinformatics analysis results of *PgLTP*

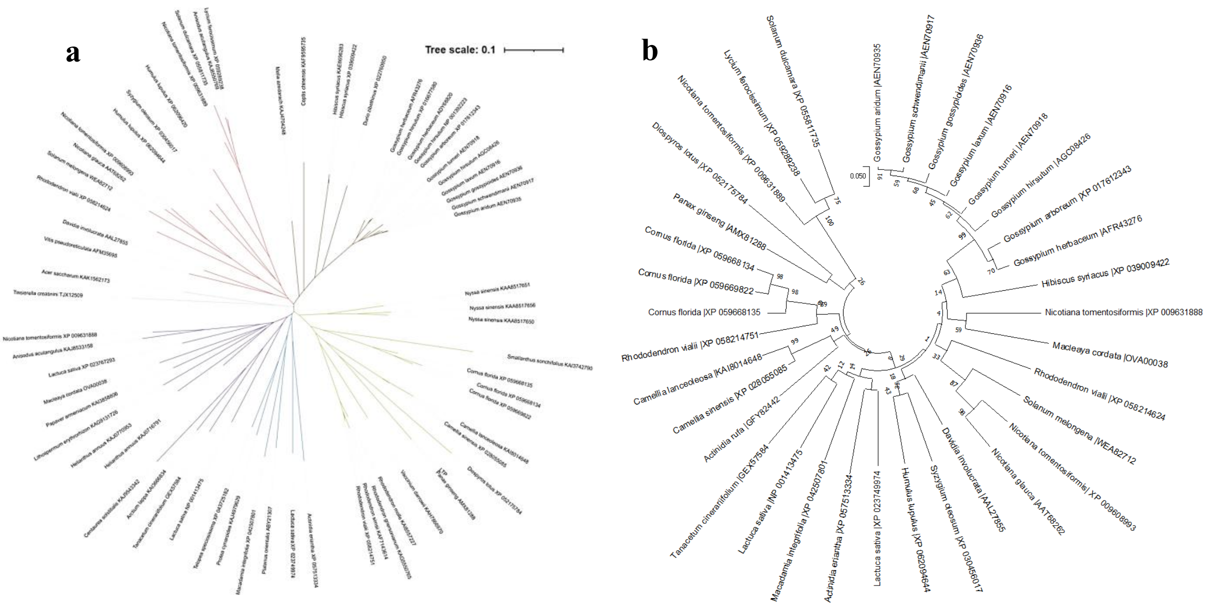

a: Phylogenetic tree constructed using DNA sequences. b: Phylogenetic tree constructed using protein sequences.

Figure S4. Analysis results of phylogenetic tree constructed by MEGA X

Table S1 Germination rate of *A. thaliana* in 1/2 MS medium containing mannitol

	Transgenic Arabidopsis				Wild-type Arabidopsis			
	0 mM	200 mM	300 mM	400 mM	0 mM	200 mM	300 mM	400 mM
1st-day	0%	0%	0%	0%	0%	0%	0%	0%
2nd-day	0%	0%	0%	0%	12%	2%	0%	0%
3rd-day	90%	0%	0%	3%	68%	12%	0%	0%
4th-day	97%	50%	3%	10%	88%	50%	2%	0%
5th-day	97%	90%	35%	18%	95%	63%	7%	2%
6th-day	98%	95%	55%	23%	97%	67%	28%	2%
7th-day	98%	95%	77%	30%	97%	70%	55%	10%
8th-day	98%	97%	82%	35%	98%	78%	63%	20%
9th-day	98%	97%	90%	45%	98%	78%	63%	38%

Table S2 qPCR amplification program

Program	Temperature	Time	Cycle
Initial Denaturation	94℃	2min	1
Denaturation	94℃	30s	
Annealing	55℃	30s	35
Extension	72℃	1min	
Final Extension	72℃	10min	1

Table S3 Primer design for qPCR amplification

Primers	Sequences 5→3
LTP-F	CATGCCATGGCTAGTTCAGCTGGA
LTP-R	CCCTCGAGCTGCACCCTTGGAGCAGT
Act-F	GGCGATGAAGCTCAATCCAAACG
Act-R	GGTCACGACCAGCAAGATCAAGACG
